# Pandemic or “Plandemic”?: The Mediating Role of Epistemic Justification Strategies in the Relationship Between COVID‐19 Conspiracy Beliefs and COVID‐19 Vaccine Conspiracy Beliefs

**DOI:** 10.1002/brb3.70275

**Published:** 2025-01-09

**Authors:** Ali Gökalp, Servet Üztemur, Po‐Ching Huang, Aslı Kartol, Hsin‐Chi Tsai, Erkan Dinç, Mark D. Griffiths, Chung‐Ying Lin

**Affiliations:** ^1^ Department of Educational Sciences Gaziantep University Gaziantep Türkiye; ^2^ Department of Turkish and Social Sciences Education, Faculty of Education Anadolu University Eskişehir Türkiye; ^3^ School of Physical Therapy, Graduate Institute of Rehabilitation Science, College of Medicine Chang Gung University Taoyuan Taiwan; ^4^ Department of Psychological Counseling and Guidance Trakya University Edirne Türkiye; ^5^ Department of Psychiatry, School of Medicine Tzu Chi University Hualien Taiwan; ^6^ Department of Psychiatry Tzu‐Chi General Hospital Hualien Taiwan; ^7^ Department of Primary Education, Faculty of Education Anadolu University Eskişehir Türkiye; ^8^ International Gaming Research Unit, Psychology Department Nottingham Trent University Nottingham UK; ^9^ Institute of Allied Health Sciences, College of Medicine National Cheng Kung University Tainan Taiwan; ^10^ Biostatistics Consulting Center, National Cheng Kung University Hospital, College of Medicine National Cheng Kung University Tainan Taiwan; ^11^ Department of Public Health, College of Medicine National Cheng Kung University Tainan Taiwan; ^12^ INTI International University Nilai Negeri Sembilan Malaysia; ^13^ Department of Physiotherapy School of Nursing and Health Studies, Hong Kong Metropolitan University Hong Kong China

**Keywords:** COVID‐19 conspiracy beliefs, COVID‐19 vaccine conspiracy beliefs, epistemic beliefs, epistemic justification, justification by authority, personal justification

## Abstract

**Background:**

In today's post‐truth times, where personal feelings and beliefs have become increasingly important, determining what is accurate knowledge has become an important skill. This is especially important during uncertainty crises (e.g., epidemics and pandemics) because alternative explanations other than scientific knowledge may be disseminated vigorously. Epistemic justification concerns how and in what way the truth of knowledge claims is justified and the criteria for knowledge to be true and/or a fact. Given this backdrop, the present study examined how individuals reacted to conspiracies in an uncertainty crisis (using the COVID‐19 pandemic as an example).

**Aim:**

The mediating role of epistemic justification was investigated regarding its relationship between COVID‐19 conspiracy beliefs and COVID‐19 vaccine conspiracy beliefs.

**Methods:**

A cross‐sectional study was conducted incorporating a multifactorial correlational design. Using convenience sampling, 690 participants (55.7% females, *M*_age_ = 32.24 years, SD = 9.75) from different regions of Türkiye completed an online survey via *Google Forms*.

**Results:**

The results demonstrated a strong and statistically significant correlation between beliefs in COVID‐19 conspiracy theories and beliefs in COVID‐19 vaccination conspiracy theories. The mediating effects of justification by authority and personal justification were statistically significant between COVID‐19 conspiracy beliefs and COVID‐19 vaccine conspiracy theories.

**Conclusion:**

Using the COVID‐19 pandemic as an example, the present results indicated the complex relationships between conspiracy beliefs and epistemic justification. The present results indicate the importance of authorities in taking early action to provide scientific evidence and information to the public to avoid individuals believing false information.

## Graphical abstract

Relationship of COVID‐19 conspiracy beliefs with vaccine conspiracy beliefs.

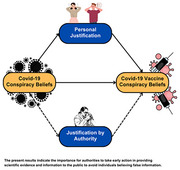

## 1 Introduction

Various conspiracy theories emerged during the COVID‐19 pandemic, such as the virus was produced in a laboratory environment (Roozenbeek et al. 2020) and that it was a man‐made biological weapon (Andrade 2020; Chen et al. 2020; Imhoff and Lamberty 2020). The number of individuals who believed in these conspiracy theories was considerably high due to the chaotic environment on social media (Juanchich et al. 2021; Freeman et al. 2022). Some of those who believed in COVID‐19 conspiracy theories did not adequately comply with the preventive rules introduced by governments worldwide in the context of the pandemic (Bierwiaczonek, Kunst, and Pich 2020; Motta, Stecula, and Farhart 2020; Pavela Banai, Banai, and Mikloušić 2022).

Many comments under the hashtag “#plandemic” on *Twitter* (now *X*), especially during the height of COVID‐19, led to the proliferation of different conspiracy theories about COVID‐19 (Kearney, Chiang, and Massey 2020). The popularity of the term “#plandemic” on social media attracted the attention of conspiracy theorists. Indeed, COVID‐19 conspiracy theories were the subject of a trilogy documentary film produced by Mikki Willis called *Plandemic* (Sethi, Roy, and Smith 2023). The movie claimed that COVID‐19 was a worldwide conspiracy to control humanity through fear. In the film, the COVID‐19 pandemic was described as an important moment in a decades‐long plan (Lytvynenko 2020). Moreover, the film claimed that COVID‐19 was engineered in a laboratory and that “Event 201,” the 2019 disaster response exercise, was a plan to release a real virus into the population (Dunlop 2020). The movie went viral shortly after circulating on the internet, with millions of views (Frenkel and Alba 2020). There was a huge increase in the number of comments made under the hashtag “#plandemic” on social media due to the movie (Kearney, Chiang, and Massey 2020). The trailer for the last movie, which spread quickly on the internet, feeding the concern about the coronavirus, was released on May 23, 2023, and the movie quickly resonated on social media. It was viewed two million times on social media channels by May 31, 2023 (Sethi, Roy, and Smith 2023).

Empirical studies have reported negative associations between COVID‐19 conspiracy beliefs and COVID‐19 health protective behaviors (Allington et al. 2021; Freeman et al. 2022). Moreover, these conspiracy beliefs were reported to play an important role in individuals' attitudes toward COVID‐19 vaccines, vaccination intentions, and antivaccination (Bertin, Nera, and Delouvée 2020; Caycho‐Rodríguez et al. 2023; Eberhardt and Ling 2021; Tuzcu and Şahin 2022; Ullah et al. 2021). Moreover, conspiracy theories about the COVID‐19 virus itself, as well as the vaccines developed against the virus, have emerged (Agley and Xiao 2021; Šrol, Čavojová, and Ballová Mikušková 2022). One prevalent conspiracy theory claimed that major pharmaceutical corporations intentionally produced the virus and subsequently produced vaccines to generate substantial financial gains. Another theory suggested that vaccines were created to manipulate human DNA (Durmuş and Ünal 2023; Hornsey, Harris, and Fielding 2018).

Given this backdrop, questions regarding the relationship between COVID‐19 conspiracy beliefs and COVID‐19 vaccine conspiracy beliefs, as well as which variables mediate the relationship between these two variables, were the subject of the present study. It is also known that past epidemics strengthen conspiracy beliefs (Bogart et al. 2010; Smallman 2015), and that an individual who believes in one conspiracy theory tends to believe in other conspiracy theories (Georgiou, Delfabbro, and Balzan 2020; Monaci 2021). Even incompatible conspiracy theories can be positively correlated (Enders, Smallpage, and Lupton 2020; Freeman et al. 2022; Miller 2020). Moreover, even if there is a contradiction between conspiracy theories, individuals can continue to believe in them. This demonstrates that conspiracy theories are predicted by a frame of mind that is inclined to believe in conspiracy theories (Uscinski and Parent 2014). It has also been reported that individuals with this mindset believed in conspiracy theories regarding COVID‐19 (Lazarević et al. 2021; Sayın and Bozkurt 2021).

Although conspiracy theories (here defined as the idea that someone or some group is acting secretly with malicious intentions; Hodapp and Von Kannon 2008) are frequently included in social and political debates, there has been an increase in empirical studies on their causes and consequences over the past two decades (Douglas et al. 2019; Hodapp and Von Kannon 2008). Associating COVID‐19 conspiracy beliefs with the conspiratorial mindset is not sufficient to understand the reasons for underlying conspiracy beliefs. Moreover, the motives underlying individuals' acceptance of conspiracy beliefs may vary. These can be psychological, demographic, political, social and/or epistemic motives (Douglas et al. 2019; Ivančík and Nováková 2023; van Prooijen and Douglas 2018).

### 1.1 Epistemic Justification as a Mediator

Epistemic justification in the present study is based on the “Epistemic and Ontological Model of Cognition” developed by Greene, Azevedo, and Torney‐Purta (2008). This model comprises two subdimensions (justification by authority and personal justification) (Greene, Torney‐Purta, and Azevedo 2010). Epistemic justification is a rational process in which the roots of knowledge are sought for how to decide what is true and what is not. The personal justification subdimension characterizes individuals who try to justify their knowledge claims based on their personal opinions or feelings and do not care much about the opinions of others in the process of justifying knowledge. In the personal justification subdimension, internal insights and intuitions are more prominent than external sources of information. The justification by the authority subdimension describes individuals who refer to a well‐respected external source (i.e., scientific papers, official sources), authority (i.e., medical doctors, academics, teachers), and their expertise to check the accuracy of their knowledge claims (Ferguson, Bråten, and Strømsø 2012; Ferguson and Braten 2013; Greene, Azevedo, and Torney‐Purta 2008; Üztemur and Dinç 2020).

Ferguson, Bråten, and Strømsø (2012) added a “justification by multiple sources” subdimension to the model based on their empirical research. This subdimension characterizes competent individuals who, in the process of making sense of multiple documents on controversial issues and sources, check and justify the consistency of information sources by cross‐checking, comparing, and verifying among various information sources, evaluating, and justifying the accuracy of information in the light of the logical framework (Ferguson et al. 2013). Therefore, it can be said that personal justification and justification by authority are on opposite sides of the spectrum in the justification of knowledge. Their negative correlation also indicates this (Ferguson and Braten 2013; Rosman et al. 2021).

Justification by authority can be characterized as an epistemic belief that relies in an absolutist way on authoritative external sources of knowledge. It emphasizes expertise and the fact that the statements of experts/scientists are seen as true. Scientists seek to extend existing research, replicate studies, criticize and interrogate old concepts, use standardized methods, and know how to deal with conflicting sources and controversial issues. Moreover, experts/scientists are more likely to share information that is not in line with conspiracy theories (Ferguson et al. 2013; Greene, Torney‐Purta, and Azevedo 2010). In other words, there could be a negative relationship between conspiracy beliefs and justification by authority (Beck et al. 2020). On the other hand, individuals often do not doubt their conspiracy beliefs and believe in them because, by nature, individuals are very likely to ignore information that contradicts their personal (internal) beliefs (Garrett and Weeks 2017).

Conspiracy beliefs are epistemically weak because of their unfalsifiable nature, and any attempt to prove them false is taken as evidence supporting the theory (Goertzel 2010). In this case, conspiracy beliefs and personal justification seem to be positively associated (Beck et al. 2020). For this reason, conspiracy beliefs have been characterized as a “paranoid style of thought” (Hofstadter 1995). Sunstein and Vermeule (2009) have even described conspiracy beliefs as a “crippled epistemology.” Conspiracy theories are effectively shielded from gaining knowledge about new information. They have a strong resistance to being questioned or undergoing renewal. Conspiracy theories rely on insufficient and feeble evidence, characterized by gaps and confusing information (Brotherton 2013). An analytical thinker who is firmly grounded in epistemic rationality is less likely to believe in conspiracy theories (Ståhl and van Prooijen 2018). Epistemic justification affects individuals' attitudes toward multiple information claims (Greene, Azevedo, and Torney‐Purta 2008). During the COVID‐19 pandemic and postpandemic, accessing accurate and reliable information has become a skill that requires mastery. Epistemic justification strategies can effectively influence decision‐making on many important issues, especially human health. In today's post‐truth times, where fake news and alternative facts are popular, a differentiated approach to scientific knowledge has become increasingly important (Beck et al. 2020). This suggests epistemic justification may affect the relationship between COVID‐19 conspiracy beliefs and COVID‐19 vaccine conspiracy beliefs.

### 1.2 The Present Study

A study associating epistemic beliefs with general conspiracy beliefs reported that individuals who rely on reasoning skills to evaluate factual claims are less likely to believe in conspiracy theories. In contrast, individuals who rely more on their intuition are more likely to support conspiracy theories (Garrett and Weeks 2017). To the best of the present authors' knowledge, very few studies have explored the relationship between epistemic justification and COVID‐19 conspiracy beliefs. More specifically, the authors are only aware of three studies. Rosman et al. (2021) reported significant negative associations between COVID‐19 vaccination intentions and personal justification and significant positive associations with justification by authority. Serrano, Crone, and Williams (2023) reported that science conspiracy beliefs and COVID‐19 conspiracy belief scores decreased as the trust in scientific research scores increased. Finally, Beck et al. (2020) reported that COVID‐19 conspiracy beliefs were positively associated with personal justification and negatively associated with justification by authority.

Given the conceptual framework and the aforementioned literature, the present study investigated the mediating effect of epistemic justification in the relationship between COVID‐19 conspiracy beliefs and COVID‐19 vaccine conspiracy beliefs. The primary expectation was that COVID‐19 conspiracy beliefs would positively predict COVID‐19 vaccine conspiracy beliefs. It was also expected that personal justification and justification by authority would mediate the positive relationship between COVID‐19 conspiracy beliefs and COVID‐19 vaccine conspiracy beliefs separately. The few empirical studies in the literature have reported associations of epistemic justification with (i) COVID‐19 conspiracy beliefs (Beck et al. 2020), (ii) trust in science (Serrano, Crone, and Williams 2023), and (iii) COVID‐19 vaccination intention (Rosman et al. 2021). However, no previous study has investigated the relationship between COVID‐19 conspiracy beliefs and COVID‐19 vaccine conspiracy beliefs and the mediating effect of epistemic justification in this relationship. Therefore, three exploratory hypotheses (H_s_) were formulated: (i) COVID‐19 conspiracy beliefs would be positively associated with COVID‐19 vaccine conspiracy beliefs (H_1_); personal justification would mediate the positive relationship between COVID‐19 conspiracy beliefs and COVID‐19 vaccine conspiracy beliefs (H_2_); and (iii) justification by authority would mediate the positive relationship between COVID‐19 conspiracy beliefs and COVID‐19 vaccine conspiracy beliefs (H_3_).

## 2 Method

A cross‐sectional study was conducted incorporating a multifactorial correlational design, which is suitable for determining potential predictive relationships between two or more variables (Fraenkel, Wallen, and Hyun 2012). Consequently, the potentially predictive effects of COVID‐19 conspiracy beliefs and epistemic justification (i.e., personal justification and justification by authority) on COVID‐19 vaccine conspiracy beliefs were examined.

### 2.1 Procedure and Participants

The study procedures were in accordance with the Declaration of Helsinki. The first author's University Social and Human Sciences Ethics Committee approved the study. Using convenience sampling, 690 participants from different regions of Türkiye completed an online survey (via *Google Forms*) between May and June 2024. The link to the form was shared on social media platforms (*Instagram*, *Facebook*, and *X*) and *WhatsApp* groups. To complete the survey, participants had to be at least 18 years old. Participants were provided with detailed information regarding the purpose of the study and ethical considerations. Participants had to read the study details and provide their informed consent to complete the survey. There were no missing data because the survey could not be submitted unless all items were answered. Participants responded to the survey anonymously and did not receive any payment. Control items (e.g., “*This item has been added for control purposes. Please tick ‘strongly agree’ on this item*”) were included to ensure participants completed the questions properly. All the participants marked “strongly agree” to this item. Information about the participants is presented in Table 1. Moreover, the total sample was randomly separated into two subsamples for psychometric testing: an exploratory factor analysis (EFA) subsample (*n* = 377) and a CFA subsample (*n* = 313).

**TABLE 1:** Participants' characteristics.

	Mean (SD) or *n* (%)		
	Total sample (*N* = 690)	EFA subsample (*n* = 377)	CFA subsample (*n* = 313)
Age	31.24 (9.75)	30.99 (9.38)	31.53 (10.19)
Gender			
Female	384 (55.7%)	205 (54.4%)	179 (57.2%)
Male	306 (44.3%)	172 (45.6%)	134 (42.8%)
Educational level			
High school	108 (16.4%)	59 (15.6%)	54 (17.3%)
Undergraduate	88 (12.8%)	45 (11.9%)	43 (13.7%)
Graduate	413 (59.9%)	233 (61.8%)	180 (57.5%)
Postgraduate	76 (11%)	40 (10.6)	36 (11.5%)

Abbreviations: CFA, confirmatory factor analysis; EFA, exploratory factor analysis.

### 2.2 Measures

The Vaccine Conspiracy Beliefs Scale (VCBS) (Shapiro et al. 2016) and the Justification for Knowing Questionnaire (JFK‐Q) (Bråten et al. 2012) were adapted into Turkish. In the context of Turkish culture, a new psychometric scale was developed (i.e., the COVID‐19 Conspiracy Beliefs Scale [CCBS]) following the principles suggested by DeVellis and Thorpe (2021): (i) determination of the construct to be measured, (ii) creation of the item pool, (iii) submission of the items to expert opinion, (iv) application, (v) validity and reliability analyses, and (vi) finalization of the scale.

#### 2.2.1 Vaccine Conspiracy Beliefs Scale

The present study used a slightly adapted Turkish version of the unidimensional seven‐item VCBS (Shapiro et al. 2016). In the first stage of the adaptation, the scale was translated into Turkish as well as changing the word “vaccine” in the scale to “COVID‐19 vaccine.” Two native Turkish‐speaking English language educators translated the items. In the second stage, the Turkish version of the adapted scale was finalized based on the opinions of experts (two Turkish language experts, two measurement and evaluation experts, and two public health academicians). In the third stage, the Turkish version of the scale was administered to a group of 20 participants. Here, the think‐aloud protocol was used to check whether there were any semantically difficult‐to‐understand expressions in the Turkish version of the adapted VCBS. Revisions were made to the Turkish version of the scale using feedback from the pilot study. In the final stage, the Turkish version of the adapted VCBS was back‐translated into English by three native Turkish‐speaking English language experts and was compared with the original VCBS. The Turkish version was found to be similar to the original VCBS in terms of semantic integrity. Using the EFA subsample (*n* = 377), the results showed a unidimensional structure (Kaiser–Meyer–Olkin [KMO] = 0.885; Barlett's *χ* ^2^ = 1259.600, df = 78; *p* < 0.001, explained variance: 52.33%), in agreement with the original scale. Confirmatory factor analysis (CFA) was applied to ensure the validity of the data obtained from the Turkish version of the VCBS with the CFA subsample (*n* = 313). The CFA results demonstrated that the original unidimensional structure of the scale was confirmed, and the fit indices were at an acceptable level: *χ* ^2^/df = 4.79, SRMR = 0.03, RMSEA = 0.07, TLI = 0.97, CFI = 0.98. Items (e.g., “*People are being deceived about the protective efficacy of COVID‐19 vaccines*”) are rated on a five‐point scale from 1 (*strongly disagree*) to 5 (*strongly agree*). The total scores range from 7 to 35, with higher scores indicating greater belief in COVID‐19 vaccine conspiracies. The internal consistency coefficients of the scale were very good (Cronbach's *α* = 0.88; McDonald's *ω* = 0.88).

#### 2.2.2 Justification for Knowing Questionnaire

The original 14‐item JFK‐Q (Bråten et al. 2012) has three subscales (justification by authority, personal justification, and justification by multiple sources). The present study used two of the subscales (i.e., justification by authority dimension [six items] and personal justification dimension [three items]) because they constitute the two extremes of the epistemic justification spectrum (Bråten, Brandmo, and Kammerer 2019; Ferguson et al. 2013; Rosman et al. 2021). Since there is no Turkish version, the JFK‐Q was adapted into Turkish. Scale items were adapted to reflect medical science. For example, the item “*Knowledge about natural sciences is only personal opinion—no facts*” was changed to “*Knowledge about medical science is only personal opinion—no facts*.” The translation procedure was identical to that of the VCBS (see above). Using the EFA subsample (*n* = 377), the results showed a two‐dimensional structure (KMO = 0.791; Barlett's *χ* ^2^ = 1167.147, df = 36; *p* < 0.001, explained variance: 56.38%), in agreement with the original scale. The CFA results (*n* = 313) indicated that the fit indices for the structure of the Turkish version of JFK‐Q were acceptable: *χ* ^2^/df = 3.26, SRMR = 0.05, RMSEA = 0.06, TLI = 0.96, CFI = 0.97. Items (personal justification: e.g., “*Everyone can have different opinions about medicine science because no completely correct answers exist*”; justification by authority: “*I believe that everything I learn in medical science class is correct*”) were rated on a five‐point scale from 1 (*strongly disagree*) to 5 (*strongly agree*). The scores obtained from the justification by authority subdimension range between 6 and 30; the scores obtained from the personal justification subdimension range between 3 and 15. Higher scores obtained in each subdimension indicate greater acceptance of the justification in the subdimension. The internal consistency coefficients of the subscales were adequate (justification by authority: Cronbach's *α* = 0.82, McDonald's *ω* = 0.82; personal justification: Cronbach's *α* = 0.64, McDonald's *ω* = 0.67).

#### 2.2.3 COVID‐19 Conspiracy Beliefs Scale

During the scale development process, an item pool of 13 items was generated using empirical findings from previous studies and related literature (Antichi, Goretzko, and Giannini 2022; Debski et al. 2022; Leibovitz et al. 2021; Pfeffer et al. 2024). The 13 items were finalized in line with the opinions of three measurement and evaluation experts and two language and grammar experts (Turkish and English). Items (see Table 2) were rated on a five‐point scale from 1 (*strongly disagree*) to 5 (*strongly agree*). The total scores range from 13 to 65, and higher scores indicate greater COVID‐19 conspiracy beliefs. As the CCBS is a newly developed scale, its psychometric properties (including factor structure) were examined before it was used to investigate the study's hypotheses. The scale's psychometric results are reported in Section 2.3.

**TABLE 2:** Factor loadings of scale items in exploratory factor analysis (EFA; *n* = 377) and confirmatory factor analysis (CFA; *n* = 313).

Subdimension	Item	EFA loading	CFA loading
Concealed facts and irrational suspicion	7. In my opinion, COVID‐19 does not exist	0.775	0.599
	5. In my opinion, COVID‐19 is a myth that forces people to vaccinate	0.684	0.781
	8. I think the waves emitted by 5G or 4.5G technology allow COVID‐19 to spread to wider areas	0.673	0.677
	13. In my opinion, COVID‐19 vaccines were created to sterilize people in the long term	0.659	0.805
	6. In my opinion, COVID‐19 was deliberately created to control people and manage them with microchips	0.631	0.878
	12. The truth about the relationship between COVID‐19 and 5G or 4.5G radiation waves is hidden from the public	0.622	0.770
	10. In my opinion, mRNA vaccines were produced to alter human DNA.	0.612	0.836
Global control	4. COVID‐19 is a virus deliberately created by global powers aiming to reduce the world population	0.896	0.638
	11. COVID‐19 was developed by states as part of a biological weapons program	0.807	0.899
	2. I think global forces invented COVID‐19 to vaccinate the entire population	0.700	0.869
	9. In my opinion, COVID‐19 was produced as a virus that does not harm children and young people to reduce the elderly population in the world	0.668	0.752
	3. I think that there are some people behind the spread of COVID‐19 all over the world	0.535	0.873
	1. I think that governments are deliberately not coming up with a cure for COVID‐19	0.440	0.621

*Note*: The item numbers were reported based on the original item number. The extraction method was principal axis factoring, and the rotation method was promax oblique rotation for EFA; maximum likelihood estimator for CFA. Factor loading values < 0.4 are not reported.

### 2.3 Statistical Analyses

The EFA subsample was used to explore the potential factor structure of the CCBS, and the CFA subsample was used to confirm the CCBS factor structure proposed by the EFA findings. The CFA subsample was also used to examine the discriminant validity of the CCBS based on the HTMT ratio method. Moreover, the total sample was used to examine the convergent validity of the CCBS and to calculate the internal consistency coefficients of all scales used in the study, and examine the present study's hypotheses using mediation analyses.

For the EFA, the 13 CCBS items were suitable for factor analysis (KMO = 0.935; Barlett's *χ* ^2^ = 2739.788, df = 21; *p* < 0.001) (Field 2024). Moreover, by using principal axis factoring and adopting the promax oblique rotation method, a two‐subdimensional structure was found (explained variance: 54.3%). The factor loadings obtained with EFA are presented in Table 2.

The first CCBS subdimension (seven items), named “concealed facts and irrational suspicion,” was characterized by conspiracy beliefs that there is some private information about COVID‐19 that was hidden from everyone. The second CCBS subdimension (six items), named “global control,” was characterized by conspiracy beliefs that COVID‐19 was deliberately created by global powers and vested interests to reduce the world population. The CFA subsample (*N* = 313) confirmed the two subdimensions. Moreover, the psychometric properties of the CCBS at the scale level were acceptable (Table 3). More specifically, the internal consistency coefficient, composite reliability (CR), and average variance extracted (AVE) of the two CCBS subscales were all good (Table 3). Internal consistency values greater than 0.7, AVE values greater than 0.50, and CR values greater than 0.60 (Fornell and Larcker 1981), support the convergent validity of the CCBS. The heterotrait–monotrait (HTMT) analysis with HTMT ratio of factor loadings less than 0.90 (Henseler, Ringle, and Sarstedt 2015) supports the discriminant validity of CCBS (HTMT = 0.86).

**TABLE 3:** Scale properties of the COVID‐19 Conspiracy Beliefs Scale (CCBS).

	CCBS	Concealed facts and irrational suspicion	Global control
Cronbach's *α* ^a^	0.930	0.890	0.886
McDonald's *ω* ^a^	0.931	0.892	0.889
Exploratory factor analysis^b^			
Eigenvalue	—	6.880	1.110
Variance explained	54.315	49.561	4.754
KMO	0.935		—
Confirmatory factor analysis^c^			
*χ* ^2^ (df)	200.720 (62)		—
*p* value	< 0.001		—
CFI	0.940		—
TLI	0.925		—
RMSEA	0.085		—
SRMR	0.043		—
IFI	0.941		
HTMT method^c^			
Concealed facts and irrational suspicion	—	1.00	
Global control	—	0.86	

Abbreviations: CFA, confirmatory factor analysis; CFI, comparative fit index; EFA, exploratory factor analysis; HTMT, heterotrait–monotrait ratio; KMO, Kaiser–Meyer–Olkin measure of sampling adequacy; RMSEA, root mean square error of approximation; SRMR, standardized root mean square residual; TLI, Tucker–Lewis index.

^a^ Based on the total sample.

^b^ Based on EFA subsample.

^c^ Based on CFA subsample.

All the data were normally distributed according to the skewness and kurtosis values. Descriptive statistics and Pearson correlation analysis were used to see how the variables studied were associated. Variance inflation factor (VIF) values were calculated to see whether there was a multicollinearity problem among the predictor variables (varying between 1.19 and 1.51). Because the calculated values were below the recommended criterion value, there was no multicollinearity problem among the predictor variables (VIF value < 4.0; Hair et al. 2006). In addition, the HTMT ratio of correlations discriminant validity was calculated using the correlation matrices of the observed variables of the Vaccine Conspiracy Beliefs and COVID‐19 Conspiracy Beliefs Scales (0.88). For discriminant validity, this value should be below 0.90 (Henseler, Ringle, and Sarstedt 2015).

Finally, the hypotheses were tested using the total sample with Model 4 (a parallel mediation model) in the PROCESS macro. The mediated effects were evaluated using 5000 bootstrapping samples at 95% confidence intervals (CIs). A mediation effect is significant when it does not contain zero between the lower limit (LL) and upper limit (UL) of the CIs (Hayes 2022). CFA, HTMT ratio, and internal consistency analyses were performed using JASP 0.18.3 (https://jasp‐stats.org/). The rest of the analyses were performed using IBM SPSS version 25.0 (IBM Corp., Armonk, NY) with the PROCESS macro plug‐in developed by Hayes (2022) being used for the mediation analysis.

## 3 Results

As seen in Table 4, the subdimensions of COVID‐19 conspiracy beliefs were positively related to COVID‐19 vaccine conspiracy beliefs and personal justification and negatively related to justification by authority. COVID‐19 vaccine conspiracy beliefs were positively associated with personal justification and negatively associated with justification by authority. There was a negative relationship between personal justification and justification by authority. All values were within the normal range (skewness and kurtosis ≤ |1.5|) recommended by Tabachnick and Fidell (2015).

**TABLE 4:** Descriptive statistics and correlation analysis (*N* = 690).

Variable	*M* ± SD	Skewness	Kurtosis	(1)	(2)	(3)	(4)	(5)	(6)
1. Global control	2.93 ± 0.88	−0.061	−0.329	—	0.759	0.502	−0.345	0.735	0.935
2. Concealed facts and irrational suspicion	2.51 ± 0.79	0.278	−0.101		—	0.535	−0.362	0.754	0.941
3. Personal justification	2.58 ± 0.75	0.377	0.521			—	−0.384	0.494	0.553
4. Justification by authority	3.17 ± 0.70	−0.228	−0.026				—	−0.447	−0.377
5. Vaccine conspiracy beliefs	3.02 ± 0.81	0.148	−0.169					—	0.794
6. COVID‐19 conspiracy beliefs	2.70 ± 0.78	0.052	−0.097						—

*Note*: All *p* < 0.01.

Abbreviations: *M*, mean; SD, standard deviation.

### 3.1 Mediation Analyses

**FIGURE 1:**
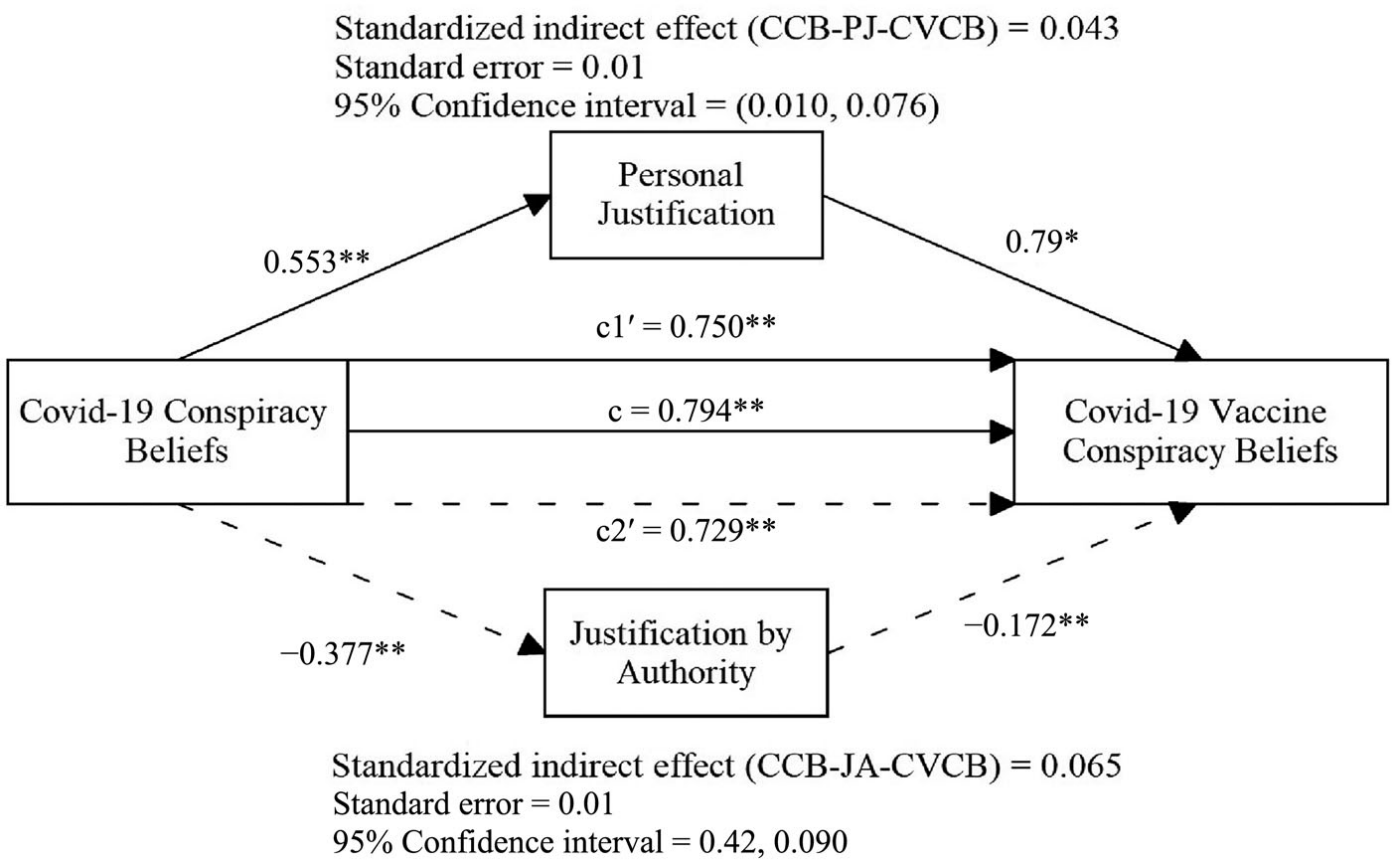
The mediating roles personal justification and justification by authority (*N* = 690). **p < 0.001, *p < 0.05. *c*, total effect; *c*1ʹ, standardized direct effect in the relationship where personal justification mediates; *c*2ʹ, standardized direct effect in the relationship where justification by authority mediates; CCB, COVID‐19 conspiracy beliefs; JA, justification by authority; PJ, personal justification.

Figure 1 shows the mediating effects of personal justification and justification by authority in the relationship between COVID‐19 conspiracy beliefs and COVID‐19 vaccine conspiracy beliefs. COVID‐19 conspiracy beliefs positively predicted COVID‐19 vaccine conspiracy beliefs; therefore, H_1_ was supported (standardized coefficient [*β*] = 0.794, *p* < 0.001). COVID‐19 conspiracy beliefs explained 63.1% of the variance in COVID‐19 vaccine conspiracy beliefs (*R* ^2^ = 0.631). Personal justification was positively predicted by COVID‐19 conspiracy beliefs (*β* = 0.553, *p* < 0.001). Moreover, 30.6% of the variance in personal justification scores was explained by COVID‐19 conspiracy beliefs. In the next stage, after personal justification was included in the model, although COVID‐19 conspiracy beliefs significantly predicted COVID‐19 vaccine conspiracy beliefs, the effect coefficient of COVID‐19 conspiracy beliefs decreased (*β* = 0.750, *p* < 0.001). Furthermore, personal justification positively predicted COVID‐19 vaccine conspiracy beliefs and this effect was statistically significant (*β* = 0.079, *p* < 0.05).

As shown in Figure 1, the indirect effect of COVID‐19 conspiracy beliefs on COVID‐19 vaccine conspiracy beliefs through personal justification (0.553 × 0.079 = 0.043) corresponded to 5% (0.043/0.794 = 0.09) of the total effect (0.715 + 0.074 = 0.794). With the inclusion of personal justification in the model, the explained variance of COVID‐19 vaccine conspiracy beliefs increased by 0.04%–63.5%. The indirect path coefficient of personal justification was significant; therefore, H_2_ was supported (*β* = 0.043, SE = 0.01, 95% CI [0.010, 0.076]).

Justification by authority was negatively predicted by COVID‐19 conspiracy beliefs (*β* = −0.377, *p* < 0.001), and COVID‐19 conspiracy beliefs explained 14.2% of the variance in justification by authority scores. After justification by authority was included in the model, although COVID‐19 conspiracy beliefs significantly predicted COVID‐19 vaccine conspiracy beliefs, the coefficient of COVID‐19 conspiracy beliefs decreased (*β* = 0.0.729, *p* < 0.001). In addition, justification by authority negatively predicted COVID‐19 vaccine conspiracy beliefs and this effect was statistically significant (*β* = −0.172, *p* < 0.001).

The indirect effect of COVID‐19 conspiracy beliefs on COVID‐19 vaccine conspiracy beliefs through justification by authority (0.377 × 0.172 = 0.064) corresponded to 8% (0.064/0.794 = 0.09) of the total effect (0.729 + 0.064 = 0.794). With the inclusion of justification by authority in the model, the explained variance of COVID‐19 vaccine conspiracy beliefs increased by 2.56% to 65.66%. The indirect path coefficient of justification by the authority was significant; therefore, H_3_ was supported (*β* = 0.065, SE = 0.01, 95% CI [0.042, 0.090]).

## 4 Discussion

The present study examined the mediating effect of epistemic justification in the relationship between COVID‐19 conspiracy beliefs and COVID‐19 vaccine conspiracy beliefs. The results indicated that COVID‐19 conspiracy beliefs positively predicted COVID‐19 vaccine conspiracy beliefs. This result is in line with previous research (e.g., Lazarević et al. 2021; Sayın and Bozkurt 2021), which found that an individual who believes in one COVID‐19 conspiracy theory is more likely to believe in another COVID‐19 conspiracy theory. Previous research has indicated that conspiracy beliefs influence individual health‐related decisions (Brotherton, French, and Pickering 2013) and may lead to antivaccination (Hornsey, Harris, and Fielding 2018; Lohiniva et al. 2014). Although vaccination is seen as the most effective way to control the spread of COVID‐19, fear of COVID‐19, and problematic social media use (Ahorsu et al. 2022; Kukreti et al. 2023; Qiao, Tam, and Li 2021;), and conspiracy beliefs about vaccines (specifically COVID‐19 vaccines) can negatively affect individuals' COVID‐19 vaccination intentions (Alshehri and Sallam 2023; Durmuş and Ünal 2023; Regazzi et al. 2023). In the context of COVID‐19, unchecked misleading information on social media channels can also positively affect vaccine hesitancy towards COVID‐19 (e.g., Gao et al. 2023; Raza et al. 2023; Romer and Jamieson 2020). To minimize vaccine hesitancy, it may be important to investigate the factors associated with (and affecting) conspiracy beliefs.

Another novel result of the present study was the inverse correlation between the justification by authority subdimension and COVID‐19 conspiracy beliefs. Individuals who gave more importance to external sources of information and trusted authority in the process of justifying knowledge tended to believe less in conspiracy theories. According to Beck et al. (2020), who reported similar results, trusting the justification of authority as a way of explaining knowledge is a strong predictor of conspiracy belief avoidance. As trust in the results of scientific research increases, general conspiracy beliefs decrease (e.g., Pivetti et al. 2021; Rutjens et al. 2018). Some studies have reported negative relationships between trust in science and COVID‐19 conspiracy beliefs (Pavela Banai, Banai, and Mikloušić 2022; Pivetti et al. 2021; Serrano, Crone, and Williams 2024). In other words, as trust in science decreases, belief in conspiracy theories increases (van Mulukom et al. 2022). However, in a study examining laypeople's epistemic beliefs about medicine (Kienhues and Bromme 2012), it was found that laypeople not only relied on the directives of experts in solving their medical problems but also had to cope with the fact that they did not have the knowledge and skills necessary to evaluate the knowledge claims of experts.

It is insufficient to rely on authority/experts to justify external sources of information alone, and it is important to support this with logical reasoning. During the COVID‐19 pandemic, experts' differing opinions on many issues naturally led to chaos and mistrust. This was an important factor in popularizing conspiracy theories among the public. From this perspective, individuals' level of trust in experts decreases in justifying the accuracy of information, and their interest in alternative explanations (e.g., conspiracy theories) increases. Examining the effects of epistemic justification on COVID‐19 vaccination intentions, Rosman et al. (2021) reported a significant positive relationship between justification by authority and intention to vaccinate. Trust in information sources plays an important role in complying with COVID‐19‐related rules. (Jovančević, Cvetković, and Milićević 2022). Therefore, individuals who make decisions based on reliable scientific knowledge have more positive attitudes towards vaccines (Čavojová, Šrol, and Ballová Mikušková 2022).

On the other hand, the present study found that participants who adopted an epistemic belief based on intuition and personal judgments in justifying knowledge claims were more likely to adopt COVID‐19 conspiracy beliefs. Personal justification is a positive predictive factor for conspiracy beliefs (Beck et al. 2020). In other words, individuals who use internal reasoning in justifying knowledge consider their knowledge and experiences more important than the opinions of field experts. They act according to their subjective judgments and tend to believe in conspiracy theories more than individuals who make external justifications. Individuals who act according to their intuitions are also more likely to support conspiracy theories (Garrett and Weeks 2017). Individuals with strong beliefs in personal justification place more value on a knowledge production process in which their personal views and opinions play a leading role (Bråten et al. 2012). This means that the scientific method is downplayed or even rejected altogether (Rosman et al. 2021). Therefore, it can be said that COVID‐19 conspiracy beliefs and lack of trust in science may negatively affect attitudes toward vaccines (Durmuş and Ünal 2023).

Rosman et al. (2021) reported a negative correlation between personal justification and vaccination intention in support of these results. To address the reasons for this negative relationship and what mediates the relationships between these two variables, the empirical findings of the present study, which demonstrated a positive relationship between personal justification and conspiracy beliefs, can be considered valuable in this respect. Therefore, it can be said that conspiracy beliefs are one of the main reasons underlying vaccine opposition. When all these findings are evaluated together, more attention can be drawn to the potential of conspiracy beliefs to influence the direction of behaviors on important issues concerning human health.

### 4.1 Limitations and Implications

The present study has some limitations. First, the results were based on self‐report data, which may have led to various types of response biases. For example, participants may have provided socially desirable responses rather than expressing their true opinions, or there may have been memory recall biases. Therefore, the issues regarding response biases should be considered when interpreting the study's findings. Second, the study used a correlational design. Therefore, causal inferences between variables cannot be determined. Moreover, the relationship between COVID‐19 conspiracy beliefs and epistemic justifications may be caused by different variables. For example, it has been reported that individuals more negatively affected economically by the COVID‐19 pandemic had higher levels of COVID‐19 conspiracy beliefs (Granados Samayoa et al. 2022). Moreover, social identities may have a positive role in the formation of COVID‐19 vaccine conspiracy beliefs and anti‐science attitudes. For example, national narcissism was found to positively predict COVID‐19 vaccine conspiracy beliefs and science rejection (Marchlewska et al. 2022).

Another study limitation was that all measures were presented to the participants in a single session. This may have caused participants with hardened and immovable theories regarding COVID‐19 or vaccines to respond to items in the scale assessing epistemic justification as if they were specifically related to COVID‐19 rather than their general way of thinking. The online survey was divided into three separate sections to avoid such concerns. Participants completed the demographic items and epistemic justification form in the first section. It was impossible to see the items in the following sections (i.e., moving to the next page in the survey) without finishing the first section. Therefore, the conspiracy belief items did not affect the participants' views on epistemic justification. In future studies, data could be collected over multiple different sessions to prevent responses to particular variables from affecting each other.

Considering these limitations, qualitative studies could be conducted to holistically evaluate conspiracy beliefs, especially those concerning COVID‐19, and the reasons underlying vaccine conspiracy beliefs. More meaningful results could also be obtained using mixed‐method research in which observations, interviews, and survey data are combined to better triangulate the data. Given that the research data were collected cross‐sectionally from a relatively small sample, it is difficult to generalize the research results to a large population. Therefore, future studies should include larger and more representative samples, preferably utilizing longitudinal designs. Also, the cross‐sectional data may not fully support the sequence of justification and conspiracy proposed in the present study's hypotheses. Therefore, an ad‐hoc analysis was conducted to examine if personal justification and justification by authority can explain factors regarding both COVID‐19 conspiracy belief and COVID‐19 vaccine conspiracy belief. The regression models showed that both personal justification and justification by authority significantly explained COVID‐19 conspiracy beliefs (*F*_(2,687)_ = 140.914, *R* ^2^ = 0.291; *p* < 0.01) and COVID‐19 vaccine conspiracy beliefs (*F*_(2,687)_ = 91.185, *R* ^2^ = 0.210; *p* < 0.01). Therefore, future longitudinal studies are warranted to clarify the sequence between COVID‐19 conspiracy beliefs, justifications, and COVID‐19 vaccine conspiracy beliefs.

Despite these limitations, the present study provided new evidence in the literature by demonstrating the mediating role of epistemic justification strategies in the relationship between COVID‐19 conspiracy beliefs and COVID‐19 vaccine conspiracy beliefs. In today's post‐modern societies, popular culture and developing technology have greatly facilitated access to information. Social media's uncontrolled and unlimited nature makes it increasingly difficult to determine which information to trust. Social media acts as a catalyst for the spread of disinformation and misinformation, especially conspiracy theories. It can cause confusion for individuals exposed to a constant flow of information. Because all knowledge cannot be based on direct experience, individuals must benefit from external sources of knowledge (social media, experts, books, the internet, etc.) (Dinç and Üztemur 2019).

Most scientific topics cannot be understood in depth by individuals, given their different levels of knowledge (Porsch and Bromme 2011). This situation further increases the importance of epistemic justification. Given that there are very few studies in the literature, the results of the present study, which shows that epistemic justification strategies have an important mediating role in justifying the truth of knowledge claims, will contribute to further research in the field. Demonstrating the effects of epistemic justification strategies on decision‐making processes with empirical evidence may lead to the creation of interventions to help individuals develop critical thinking and questioning skills. It is hoped that understanding the epistemic beliefs adopted in the justification of knowledge will help stakeholders deal with conspiracy theories in the context of the COVID‐19 pandemic (and COVID‐19 vaccines in particular) and in matters that significantly affect human health in general.

## 5 Conclusion

Despite the aforementioned limitations, the strength of the present study is that the findings are consistent with theoretical explanations and the few empirical studies on the potential effects of epistemic justifications on COVID‐19 conspiracy beliefs. It is hoped that the present study's novel findings will guide future studies because it is the first study to empirically show how epistemic beliefs adopted in the justification process of knowledge appear to play a role between COVID‐19 conspiracy beliefs and COVID‐19 vaccine conspiracy beliefs. The results of the present study can be considered valuable in terms of demonstrating how the level of trust that individuals have in scientists and scientific research affects their behavior on issues that closely affect their lives (e.g., the COVID‐19 pandemic). Finally, another important finding in the study is that belief in one conspiracy more likely results in beliefs of another.

## Author Contributions

**Ali Gökalp**: conceptualization, data curation, investigation, methodology, resources, software, validation, visualization, writing–original draft, writing–review and editing. **Servet Üztemur**: conceptualization, data curation, formal analysis, investigation, methodology, resources, software, supervision, validation, visualization, writing–original draft, writing–review and editing. **Po‐Ching Huang**: conceptualization, investigation, methodology, software, validation, writing–review and editing. **Aslı Kartol**: formal analysis, investigation, methodology, resources, validation, visualization, writing–review and editing. **Hsin‐Chi Tsai**: conceptualization, investigation, methodology, software, validation, visualization, writing–review and editing. **Erkan Dinç**: formal analysis, resources, methodology, validation, visualization, writing–review and editing. **Mark D. Griffiths**: methodology, software, supervision, validation, visualization, writing–review and editing. **Chung‐Ying Lin**: conceptualization, formal analysis, investigation, methodology, software, supervision, validation, visualization, writing–original draft, writing–review and editing.

## Disclosures

The authors have nothing to report.

## Ethics Statement

All procedures performed in studies involving human participants were performed adhering to the ethical standards of the institutional and/or national research committee and with the 1964 Helsinki Declaration and its later amendments or comparable ethical standards. Ethical approval was granted by the Gaziantep University Ethics Committee (Ethics Number: 493330).

## Consent

Informed consent was obtained from all individual participants included in the study.

## Conflicts of Interest

The authors declare no conflicts of interest.

### Peer Review

The peer review history for this article is available at https://publons.com/publon/10.1002/brb3.70275.

## Data Availability

The data from the present study are available from the co‐corresponding authors upon reasonable request.
